# Proteomic profiling reveals a signature for optimizing prognostic prediction in Colon Cancer

**DOI:** 10.7150/jca.50630

**Published:** 2021-02-22

**Authors:** Zezhi Shan, Dakui Luo, Qi Liu, Sanjun Cai, Renjie Wang, Yanlei Ma, Xinxiang Li

**Affiliations:** 1 Department of Colorectal Surgery, Fudan University Shanghai Cancer Center, Shanghai 200032, China.; 2 Department of Oncology, Shanghai Medical College, Fudan University, Shanghai 200032, China.

**Keywords:** proteomic profiling, colon cancer, prognosis

## Abstract

Previous studies developed prognostic signatures largely depended on transcriptome profiles. The purpose of our present study was to develop a proteomic signature to optimize the evaluation of prognosis of colon cancer patients. The proteomic data of colon cancer patient cohorts were downloaded from The Cancer Proteome Atlas (TCPA). Patients were randomized 3:2 to train set and internal validation set. Univariate Cox regression and lasso Cox regression analysis were performed to identify the prognostic proteins. A four-protein signature was developed to divide patients into a high-risk group and low-risk group with significantly different survival outcomes in both train set and internal validation set. Time-dependent receiver-operating characteristic at 1 year demonstrated that the proteomic signature presented more prognostic accuracy [area under curve (AUC = 0.704)] than the American Joint Commission on Cancer tumor-node-metastasis (AJCC-TNM) staging system (AUC = 0.681) in entire set. In conclusion, we developed a proteomic signature which can improve prognostic accuracy of patients with colon cancer and optimize the therapeutic and follow-up strategies.

## Introduction

Colon cancer is one of the most common malignancies worldwide 1. Radical surgery alone or combined with adjuvant chemotherapy is the standard regimen for management of colon cancer without distant metastasis. However, about 25%-40% patients will suffer recurrence and metastasis after receiving standardized treatment 2. The early detection and management of relapse contribute to improve prognosis. To date, prognostic prediction is largely depended on the tumor, lymph node, metastasis (TNM) staging system 3. On this basis, novel prognostic models have been developed to improve prognostic prediction and optimize the therapeutic and follow-up strategies using clinicopathologic and genetic factors 4, 5. Notably, with the advance of genome-sequencing technologies, gene signatures at mRNA level presented an excellent prediction of colon cancer prognosis 6-8. However, only limited studies developed signatures at the level of protein to guide patients' prognostic stratification 9, 10.

Mass spectrometry-based proteomics can detect global protein abundance and post-translational modifications and provide comprehensive biological perspectives, which could not be replaced by genomic analysis alone 11, 12. In the present study, we identified robust prognostic proteins and constructed a proteomic classifier in colon cancer using The Cancer Proteome Atlas (TCPA) database. To our best knowledge, the proteomic signatures have not been reported in colon cancer previously.

## Materials and Methods

The proteomic data (Level 4) of colon cancer patient cohorts (COAD) were downloaded from The Cancer Proteome Atlas (TCPA) (https://www.tcpaportal.org). Package impute (Bioconductor) was applied to impute the missing values. The clinical data were downloaded from The Cancer Genome Atlas (TCGA) database (https://portal.gdc.cancer.gov).

### Development and validation of a proteomic signature

Firstly, patients were randomized 3:2 to train series and internal validation series. Univariate Cox regression analysis was performed to identify the biomarkers for prognosis in train set. Proteins with significant differences (p<0.05) were selected for LASSO Cox regression model. Finally, a multivariate Cox regression analysis was conducted to construct a multi-protein-based classifier for prognostic prediction of colon cancer patients. According to specific risk score formula, patients were divided into high-risk and low-risk groups with significantly different survival outcomes using the median value of the train series as the cutoff point.

### Statistical analysis

Kaplan-Meier curve was depicted to compare survival differences between high-risk group and low-risk group. Multivariate Cox regression analysis was conducted to identify independent prognostic factors. Receiver-operating characteristic (ROC) curve was plotted to evaluate the prognostic or predictive accuracy of the proteomic signature and clinicopathological factors. All statistical analyses were performed with R (version 3.6.1, https://www.r-project.org/).

## Results

### Development of a proteomic signature from the train set

**Figure 1:**
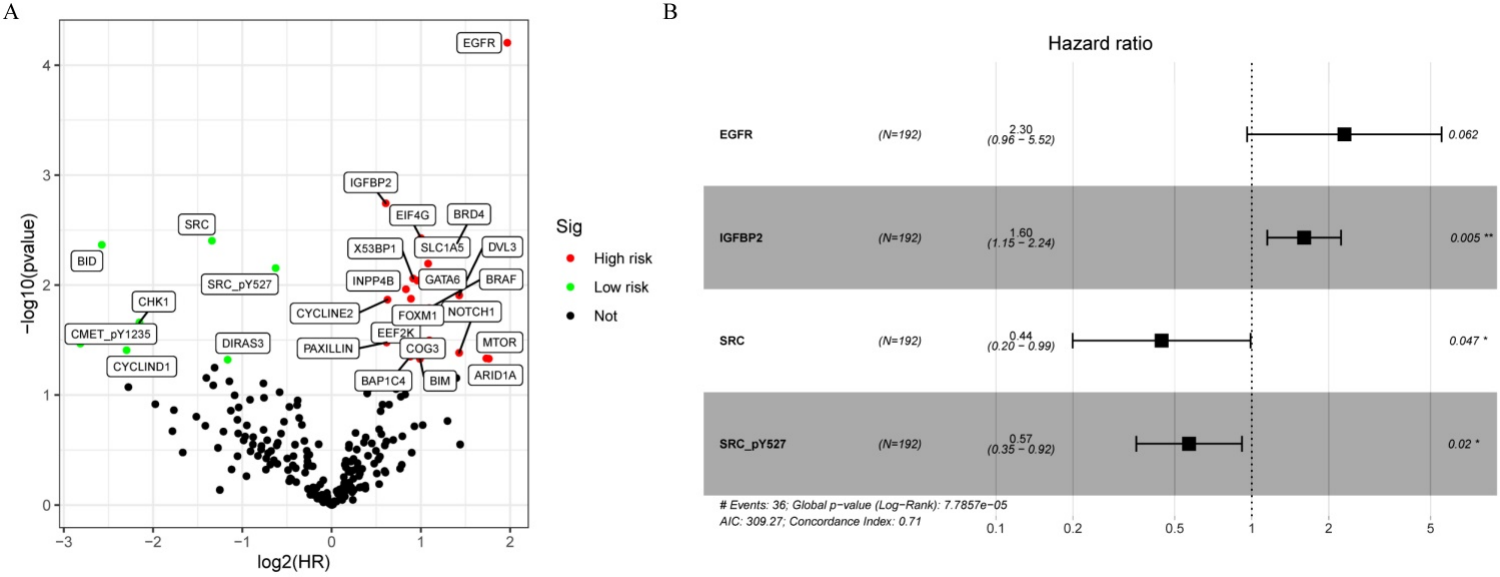
Development of a proteomic signature. A. Volcano plot of univariate Cox regression analysis. B. Forest plot of the multivariate Cox regression analysis.

**Figure 2:**
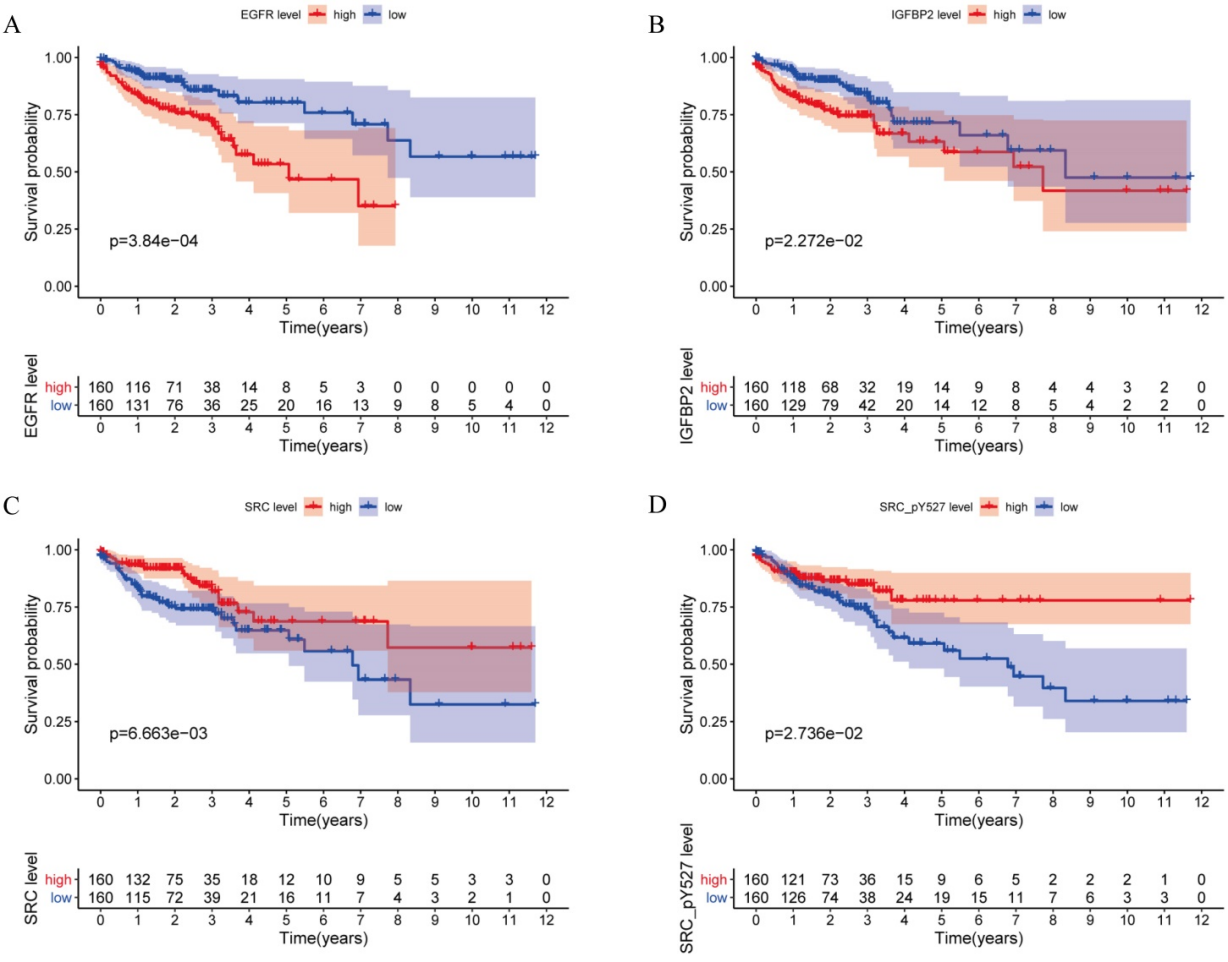
Kaplan-Meier curves for EGFR, IGFBP2, SRC and SRC_pY527.

A total of 315 colon cancer patients with complete proteomic profiling and survival data were included in our study. Patients were randomized 3:2 to train set and internal validation set. Twenty-five robust prognostic proteins were identified using univariate Cox regression analysis (Figure 1A). Lasso Cox regression and stepwise multivariate Cox regression were performed to construct a proteomic signature. Finally, a four-protein signature was developed and forest plot was presented (Figure 1B). The risk score = (0.834071775625132 × expression level of EGFR) + (0.471960975428313 × expression level of IGFBP2) + (-0.810781951083818 × expression level of SRC) + (-0.563796255046605 × expression level of SRC_pY527). Kaplan-Meier curves were plotted for each protein using the median value of the protein as the cutoff point. High expression of EGFR or IGFBP2 was associated with poor prognosis while high expression of SRC or SRC_pY527 predicted superior survival in colon cancer (Figure 2).

### The prognostic value of the four-protein signature in train set and internal validation set

**Figure 3:**
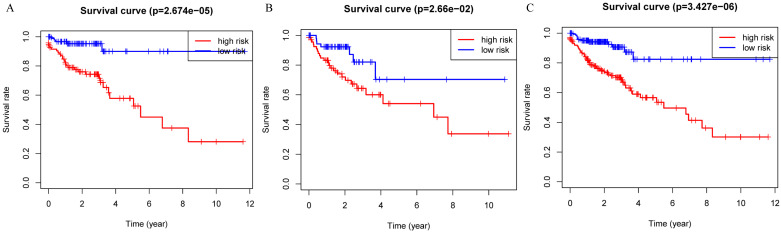
Kaplan-Meier curves for the proteomic signature. A. Train set; B. Internal validation set; C. Entire set.

**Figure 4:**
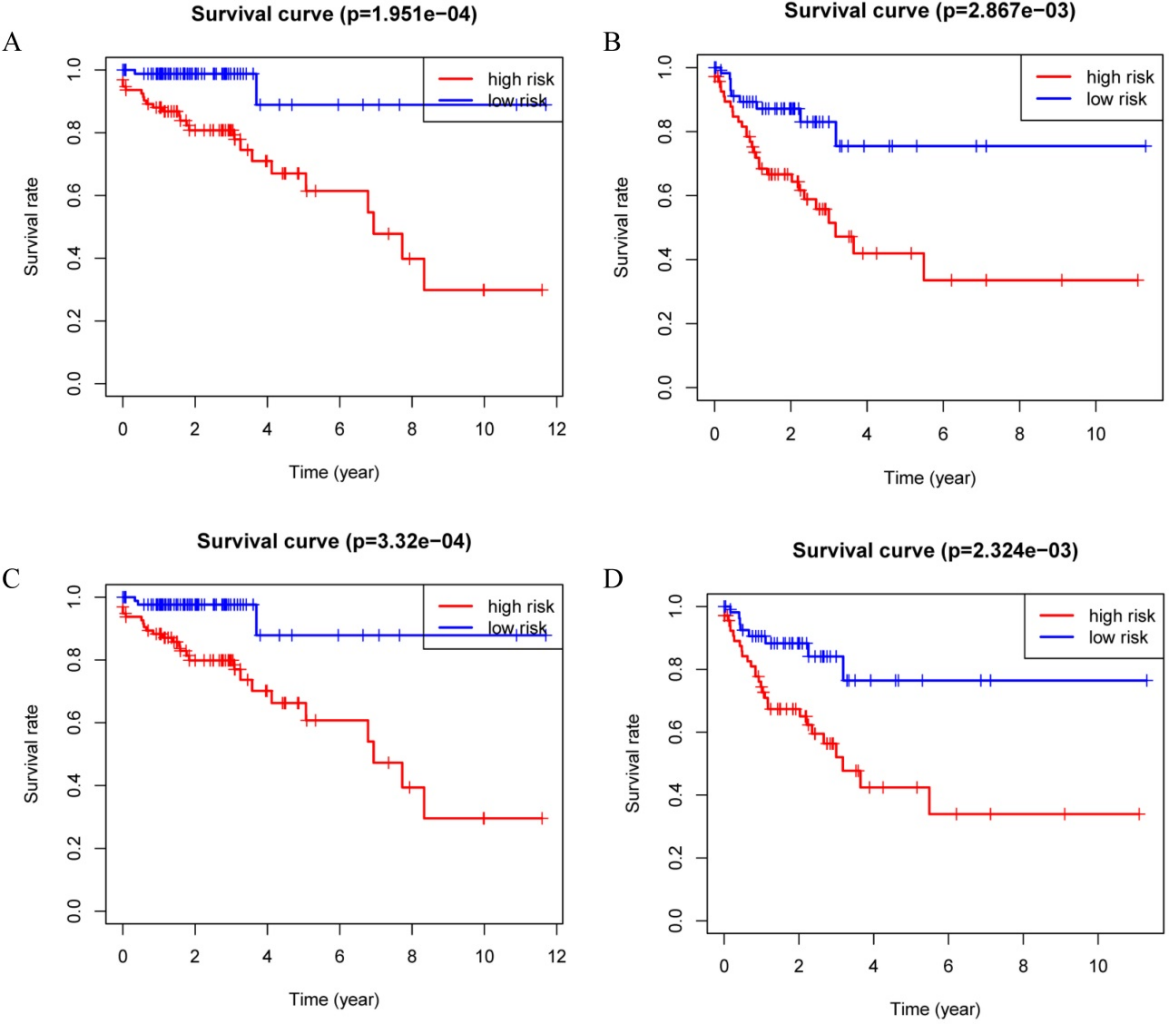
Kaplan-Meier curves for the proteomic signature in subgroups. A. Stage I and stage II; B. Stage III and stage IV; C. Lymph node negative; D. Lymph node positive.

Patients were divided into a low-risk group and high-risk group using the median risk score as the cutoff value. Patients with higher risk scores had a worse prognosis as compared to those with lower risk scores in train set, internal validation set and entire set (Figure 3). Stratified analysis revealed that the four-protein signature still had prognostic values in stage I+II, stage III+IV, lymph node positive and lymph node negative subgroups (Figure 4). The distribution of the proteomic risk score, the survival status of patients and heatmap of the proteomic expression profiles were also presented (Figure S1, S2 and S3).

### Independence and accuracy of the proteomic signature in predicting prognosis

**Figure 5:**
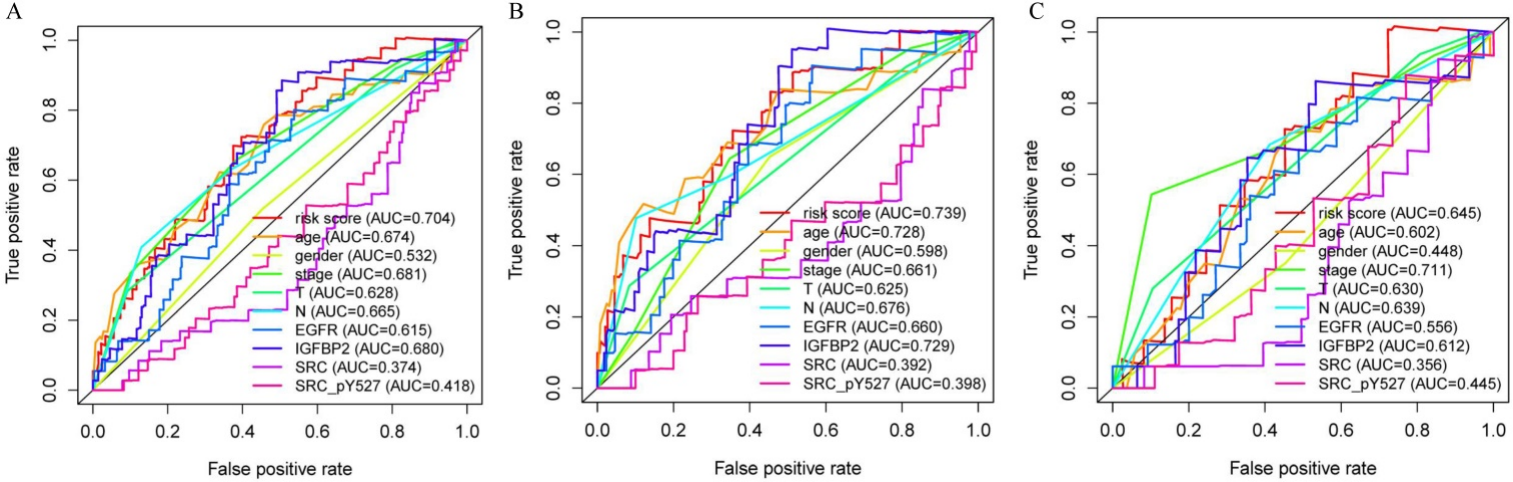
Receiver operating characteristic (ROC) analysis of the sensitivity and specificity of the proteomic signature, each protein and clinicopathological features. A. Entire set; B. Train set; C. Internal validation set.

Multivariate analysis showed that our proteomic signature remained an independent prognostic factor in entire set (Table 1). Clinicopathological characteristics of colon cancer patients in TCPA database were detailed in Table S1. Additionally, we performed ROC analysis to compare the sensitivity and specificity of prognostic prediction among proteomic signature, single protein, age, gender, T stage, N stage and AJCC stage. Time-dependent receiver-operating characteristic at 1 year demonstrated that the proteomic signature presented more prognostic accuracy [area under curve (AUC = 0.704)] than the American Joint Commission on Cancer tumor-node-metastasis (AJCC-TNM) staging system (AUC = 0.681) in entire set (Figure 5A). The AUC values in train and internal validation set were also presented and compared (Figure 5B, 5C).

### Constructing protein co-expression networks

**Figure 6:**
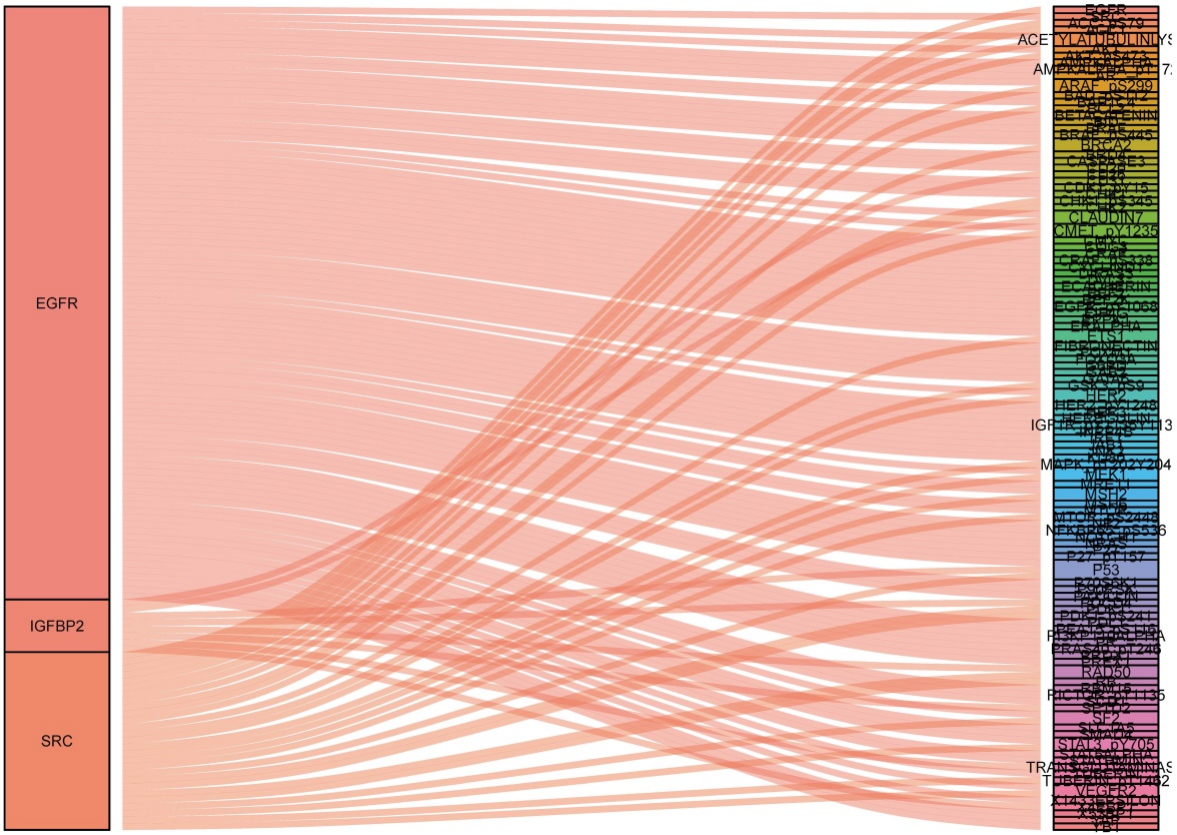
Sankey diagram of the correlations between proteins.

To identify what proteins were significantly associated with the expression of EGFR, IGFBP2, SRC and SRC_pY527 (R>0.2 or R<-0.2, p<0.05), sankey diagram was plotted (Figure 6). EGFR had more co-expressive proteins than the others.

## Discussion

Integrated transcriptome profiling of colon cancer has increased our knowledge of molecular features relevant to carcinogenesis. A number of studies developed and validated multigene signatures to predict prognosis based on global mRNAs profiling. Recently, global proteomic data which provided novel insights into the comprehensive understanding of cancers, have become focus of attention 13. Compared with a single proteomic biomarker, the combination of the prognostic proteins may have better predictive efficacy.

In this study, we established a novel proteomic signature (including EGFR, IGFBP2, SRC and SRC_pY527) for prognostic prediction of colon cancer using TCPA database. The survival curves revealed a significant separation between low-risk and high-risk patients in both training set and internal validation set. Stratified by AJCC stage and lymph node status, the proteomic signature remained an excellent prognostic model. Time-dependent ROC at 1 year demonstrated that our signature had the most significant accuracy in predicting prognosis as compared to other indicators, indicating that the risk model developed from the four proteins could be a useful tool for colon cancer survival prediction.

Epidermal growth factor receptor (EGFR), a member of the subclass I of the receptor tyrosine kinase super-family, is overexpressed in 49% to 82% of colorectal cancer 14-16. EGFR is one of the most promising targets for the management of metastatic colorectal cancer. However, EGFR testing for colorectal cancer patients has no predictive value of response to EGFR inhibitors 17, 18. The RAS/RAF/MAPK pathway is downstream of EGFR. Evidence indicated that RAS and BRAF status had predictive value of response to cetuximab or panitumumab therapy 19-21.

Insulin-like growth factor-binding protein 2 (IGFBP2) is a member of the IGFBP family that bind IGFs with high affinity 22. Previous studies suggested that IGFBP2 expression was upregulated in multiple tumors 23-25. Recently, Liu et al reported that IGFBP2 promoted vasculogenic mimicry formation via targeting CD144 and MMP2 expression in glioma 26. Gao et al indicated that IGFBP2 could drive epithelial-mesenchymal transition and invasive character by activating the NF-κB pathway in pancreatic ductal adenocarcinoma 27. Nevertheless, the function and mechanisms of IGFBP2 in colon cancer remains unclear. Our study revealed that high expression IGFBP2 indicated poor prognosis.

Proto-oncogene tyrosine-protein kinase Src contains an SH3 domain, an SH2 domain, a protein-tyrosine kinase domain, and a regulatory tail and participates in multiple biological processes 28. Src could be activated by upstream signaling pathways to form phospho-Src (p-Src), and p-Src could activate downstream signaling pathways by phosphorylating the target proteins 29, 30. Recent studies suggested that Src family kinases were involved in carcinogenesis. Hu et al demonstrated that the expression of Src and p-Src was significantly upregulated in osteochondroma and could be used as robust indicators to predict prognosis 31. Singh et al found that Src and p-Src could promote colon cancer invasion and metastasis 32. Intriguingly, our study indicated that high expression of SRC and SRC_pY527 was associated with superior prognosis in colon cancer patients. Further precise regulation mechanisms of SRC and SRC_pY527 in colon cancer are needed to be explored.

Finally, we also identified several proteins which significantly associated with the expression of EGFR, IGFBP2, SRC and SRC_pY527. These proteins should also be further explored. Several limitations are needed to address in our present study. Firstly, only over two hundred proteins were identified in TCPA database, the information about a lot of critical proteins were missing. Secondly, the lack of external validation resulted in limited clinical value of our signature. Further research regarding its external validation and clinical utility are needed. Lastly, molecular biology experiments are necessary for clarifying the underlying molecular mechanism of our proteomic signature.

In conclusion, our study established a novel proteomic signature for improving prognostic prediction in colon cancer, which may assist to develop individual therapeutic and follow-up strategies.

## Supplementary Material

Supplementary figures.

## Figures and Tables

**Table 1:** Univariable and multivariable Cox regression analysis in colon cancer

Variable	Univariate analysis		Multivariate analysis	
	HR (95% CI)	*P*	HR (95% CI)	*P*
Proteomic signature	1.207 (1.122-1.298)	<0.001	1.158 (1.070-1.254)	<0.001
Age	1.053 (1.025-1.081)	<0.001	1.053 (1.026-1.080)	<0.001
Gender	0.913 (0.558-1.493)	0.717	1.095 (0.658-1.820)	0.727
Stage	1.957 (1.474-2.598)	<0.001	2.201 (1.629-2.975)	<0.001
