# Supplementary material for: Proteomic profiling reveals a signature for optimizing prognostic prediction in Colon Cancer
Source: J Cancer. 2021 Feb 22;12(8):2199–205. doi: 10.7150/jca.50630 (PMC7974900; doi:10.7150/jca.50630)

Supplementary Figure 1 The proteomic signature risk score distribution. A. Train set. B. Internal validation set.

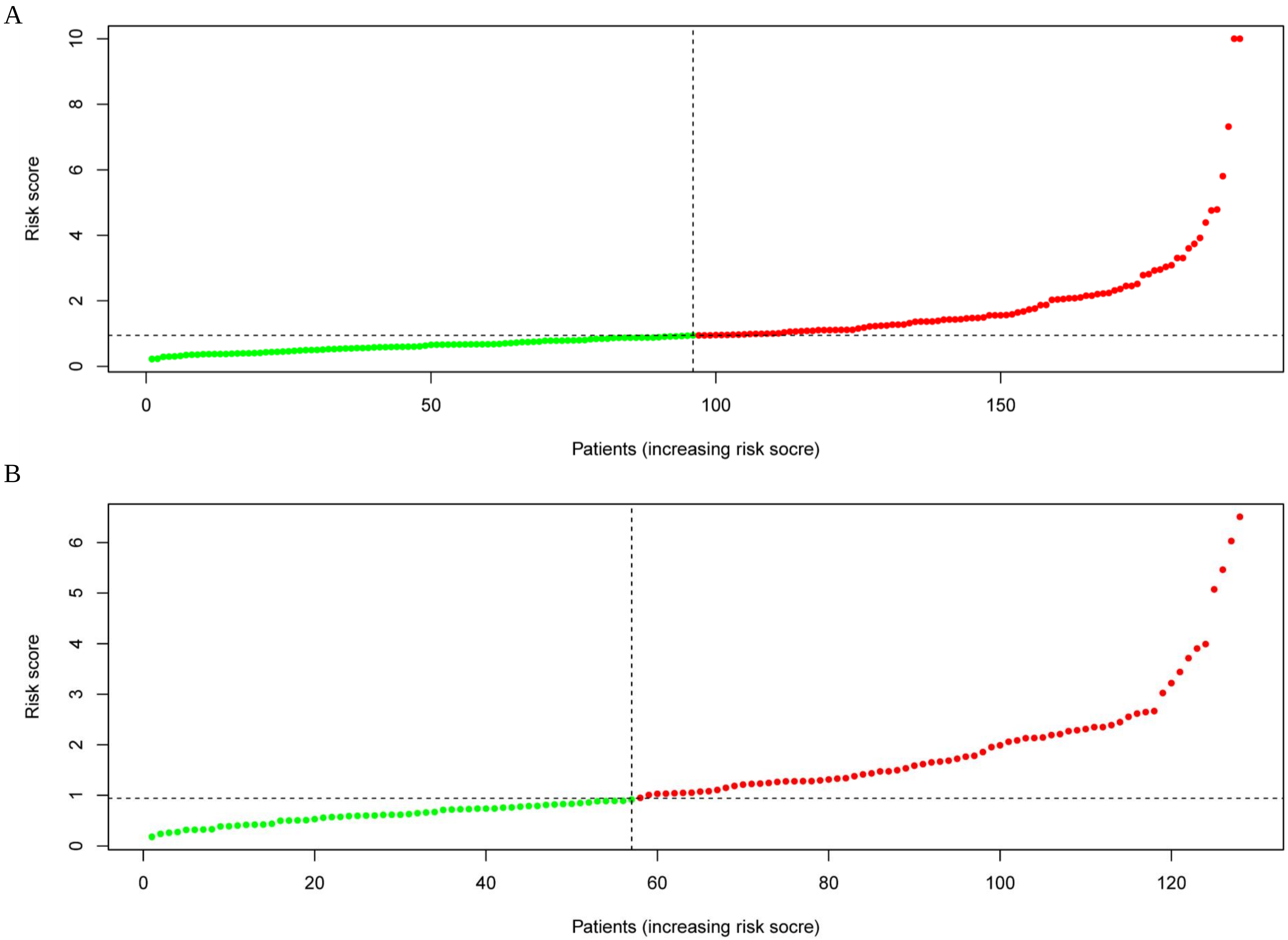

Supplementary Figure 2 The distribution of patients' survival status and time. A. Train set. B. Internal validation set.

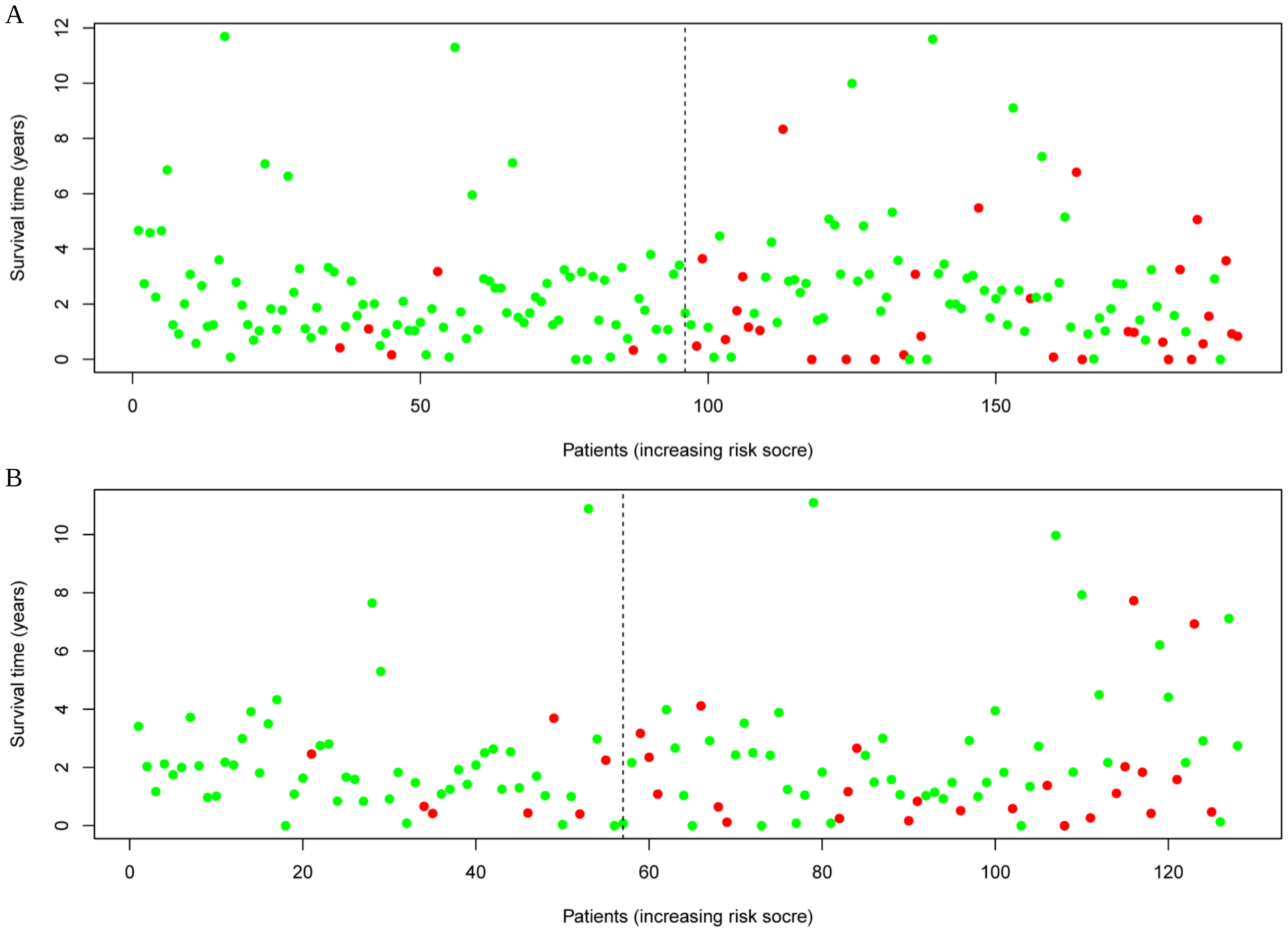

Supplementary Figure 3 Heatmap of the proteomic expression profiles. A. Train set. B. Internal validation set.

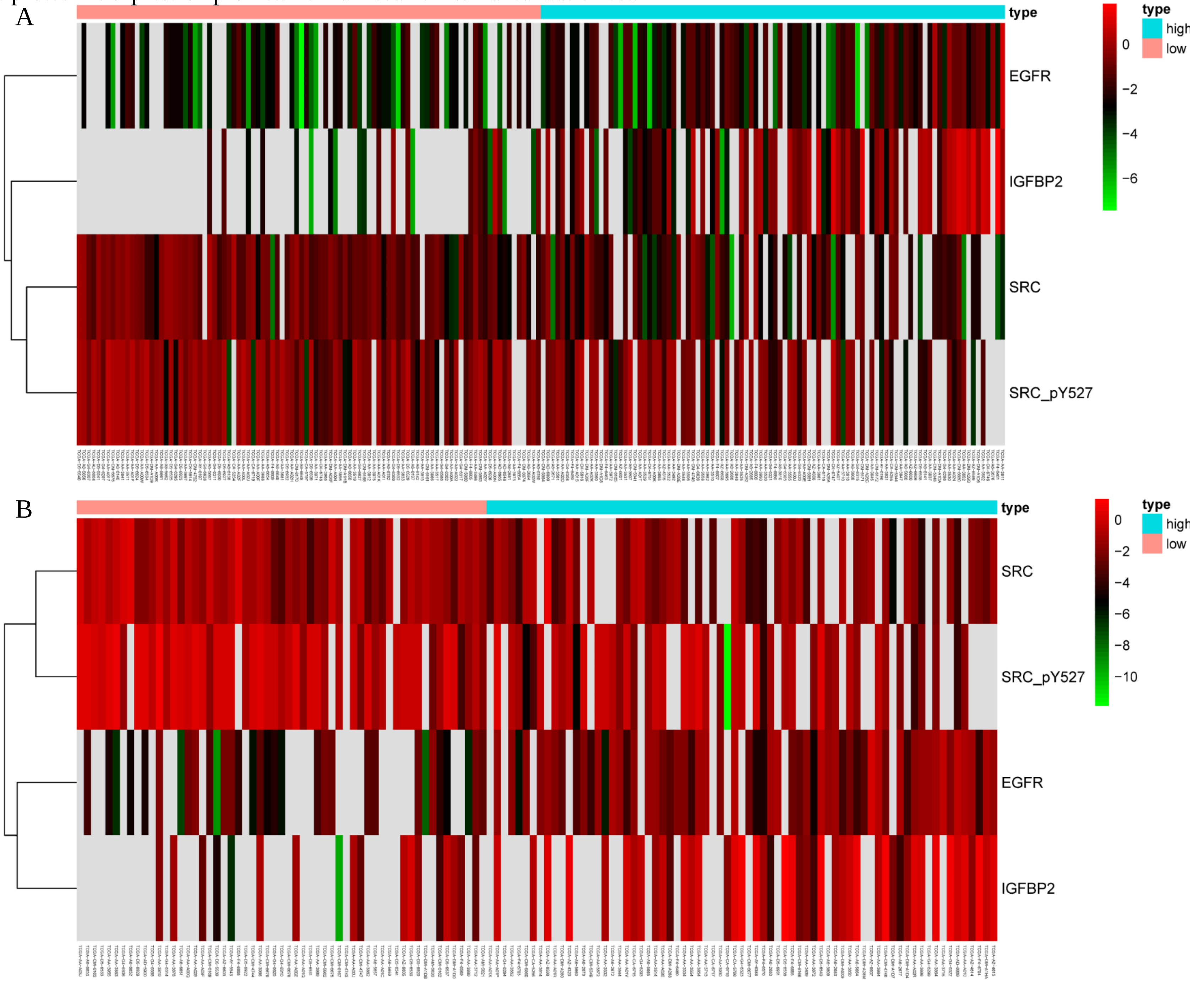

Supplement: Supplementary file 1 — Supplementary figures. [file jcav12p2199s1.pdf]
